# Benchmarking and integrating genome-wide CRISPR off-target detection and prediction

**DOI:** 10.1093/nar/gkaa930

**Published:** 2020-11-02

**Authors:** Jifang Yan, Dongyu Xue, Guohui Chuai, Yuli Gao, Gongchen Zhang, Qi Liu

**Affiliations:** Translational Medical Center for Stem Cell Therapy and Institute for Regenerative Medicine, Shanghai East Hospital, Bioinformatics Department, School of Life Sciences and Technology, Tongji University, Shanghai 200092, China; Translational Medical Center for Stem Cell Therapy and Institute for Regenerative Medicine, Shanghai East Hospital, Bioinformatics Department, School of Life Sciences and Technology, Tongji University, Shanghai 200092, China; Translational Medical Center for Stem Cell Therapy and Institute for Regenerative Medicine, Shanghai East Hospital, Bioinformatics Department, School of Life Sciences and Technology, Tongji University, Shanghai 200092, China; Translational Medical Center for Stem Cell Therapy and Institute for Regenerative Medicine, Shanghai East Hospital, Bioinformatics Department, School of Life Sciences and Technology, Tongji University, Shanghai 200092, China; Translational Medical Center for Stem Cell Therapy and Institute for Regenerative Medicine, Shanghai East Hospital, Bioinformatics Department, School of Life Sciences and Technology, Tongji University, Shanghai 200092, China; Translational Medical Center for Stem Cell Therapy and Institute for Regenerative Medicine, Shanghai East Hospital, Bioinformatics Department, School of Life Sciences and Technology, Tongji University, Shanghai 200092, China

## Abstract

Systematic evaluation of genome-wide Clustered Regularly Interspaced Short Palindromic Repeats (CRISPR) off-target profiles is a fundamental step for the successful application of the CRISPR system to clinical therapies. Many experimental techniques and *in silico* tools have been proposed for detecting and predicting genome-wide CRISPR off-target profiles. These techniques and tools, however, have not been systematically benchmarked. A comprehensive benchmark study and an integrated strategy that takes advantage of the currently available tools to improve predictions of genome-wide CRISPR off-target profiles are needed. We focused on the specificity of the traditional CRISPR SpCas9 system for gene knockout. First, we benchmarked 10 available genome-wide off-target cleavage site (OTS) detection techniques with the published OTS detection datasets. Second, taking the datasets generated from OTS detection techniques as the benchmark datasets, we benchmarked 17 available *in silico* genome-wide OTS prediction tools to evaluate their genome-wide CRISPR off-target prediction performances. Finally, we present the first one-stop **i**ntegrated **G**enome-**W**ide **O**ff-target cleavage **S**earch platform (*iGWOS*) that was specifically designed for the optimal genome-wide OTS prediction by integrating the available OTS prediction algorithms with an AdaBoost ensemble framework.

## INTRODUCTION

The lack of comprehensive investigations of Clustered Regularly Interspaced Short Palindromic Repeats (CRISPR) on-target efficacy (sensitivity) and off-target profiles (specificity) has hindered successful application of the CRISPR system for clinical therapies. The CRISPR on-target single guide RNA (gRNA) design and efficacy prediction have been extensively studied and benchmarked (1). Many genome-wide high-throughput experimental techniques and *in silico* tools have also been proposed for detecting and predicting genome-wide CRISPR off-target profiles. Although these techniques and tools have been evaluated in several studies (2–6), the evaluations were not performed in a systematic, comprehensive, and objective manner. Several main challenges remain: (i) the genome-wide CRISPR off-target profile detection techniques have not been systematically benchmarked; (ii) previous comparisons of CRISPR off-target prediction tools were not comprehensive from a genome-wide perspective; (iii) while genome-wide off-target predictions are expected to be boosted in an aggregated way by carefully integrating the available prediction tools, this aggregation of tools is yet to be explored.

To this end, we present a comprehensive study that benchmarks the available genome-wide off-target cleavage site (OTS) detection techniques as well as the *in silico* OTS prediction tools. The first benchmark of genome-wide OTS detection techniques will provide objective knowledge and benchmark datasets to be utilized in the following benchmark of genome-wide *in silico* OTS prediction tools; therefore, these two benchmarks can be performed sequentially. Furthermore, we also present the first one-stop **i**ntegrated **G**enome-**W**ide **O**ff-target cleavage **S**earch platform(*iGWOS*) that was designed specifically for the optimal OTS prediction by integrating the available OTS prediction algorithms with an AdaBoost ensemble learning model.

## MATERIALS AND METHODS

**Figure 1.**
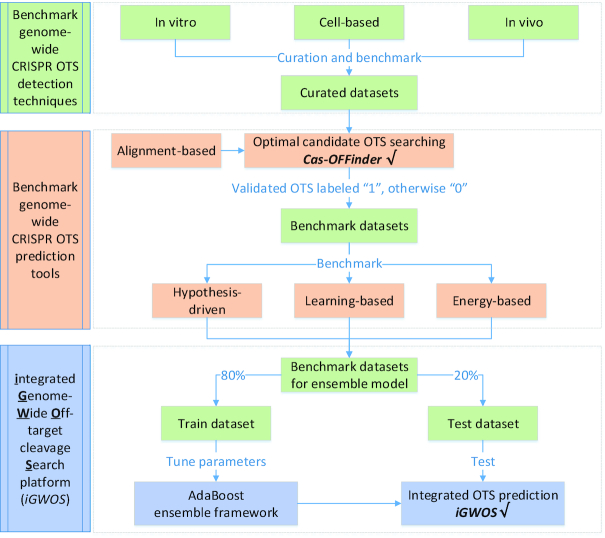
The overall workflow of our benchmark study. We benchmarked the genome-wide CRISPR OTS detection techniques and *in silico* off-target cleavage site (OTS) prediction tools in a sequential approach, and finally present the *iGWOS* platform for the integrated OTS prediction with an Adaboost ensemble learning model.

Figure 1 presents the overall workflow for benchmarking genome-wide CRISPR OTS detection and prediction in a sequential manner. The benchmark of experimental CRISPR OTS detection techniques and *in silico* prediction tools, as well as the development of the *iGWOS* platform are presented in the following sections.

### Benchmark genome-wide CRISPR OTS detection techniques

#### Categories of experimental CRISPR OTS detection techniques

Table 1 presents 10 available genome-wide double-strand breaks (DSBs) capture techniques that were developed to detect Cas9-induced DSBs in human species. They are categorized into three types: (i) *in vitro* techniques, in which the CRISPR-Cas9 system induces DSB cleavage sites in the purified genomic DNA, including *CIRCLE-seq* (7), *Digenome-seq* (8,9), *DIG-seq* (10) and *SITE-Seq* (11); (ii) cell-based techniques, in which Cas9 induces cleavage sites in living cells, including *BLESS* (12,13), *GUIDE-seq* (14), *HTGTS* (15,16), *IDLV* capture (17), and *PEM-seq* (18) and (iii) *in vivo* techniques, i.e. *DISCOVER-Seq* (19), in which Cas9 induces cleavage sites *in vivo*.

**Table 1.** Overview of three categories of genome-wide OTS detection techniques benchmarked in our study

Category	Technique	Reported sensitivity	Reference
*In vitro*	CIRCLE-seq	<0.0017%	(7)
	Digenome-Seq	0.10%	(8,9)
	DIG-seq	0.10%	(10)
	SITE-Seq	Concentration-dependent	(11)
Cell-based	BLESS	Not quant.	(12)
	GUIDE-seq	0.10%	(14)
	HTGTS	Not quant.	(16)
	IDLV capture	0.04–0.5%	(17)
	PEM-seq	Not quant.	(18)
*In vivo*	DISCOVER-Seq	0.30%	(19)

#### Curation of OTS datasets from individual OTS detection techniques

We first comprehensively summarized the 10 available genome-wide OTS detection techniques, and collected 11 genome-wide OTS datasets generated from the available 10 detection techniques (with two datasets generated from *Digenome-seq*). Then we applied the curation of gRNAs and their corresponding OTS detected from each dataset. Hg19 was taken as the reference genome, so the genomic coordinates based on hg38 in the *Digenome-seq* and *SITE-Seq* datasets were converted to hg19by tool *LiftOver* from UCSC genome browser (20). The benchmark study focused on the traditional CRISPR SpCas9 system and utilized gRNAs with 20 nucleotides (nt) followed by NGG-PAM. Our primary curation indicated that a high GC-content gRNA ‘GACCCCCTCCACCCCGCCTCCGG’ (with 80% GC content) targeting gene *VEGFA*, had a considerable proportion of OTS detected by *CIRCLE-seq* (2499/6903), *Digenome-seq* (21/138), *DISCOVER-Seq* (56/58) and *GUIDE-seq* (150/403). Previous studies also indicated that it is difficult to target GC-rich genes with gRNAs and that high GC% gRNAs tend to have weak specificity (2,21). Therefore, gRNAs with a GC content higher than 75% were excluded from our datasets. After carefully screening the OTS from these datasets, we noticed that a small portion of OTS detected by *BLESS* (3/17), *GUIDE-seq* (1/403), and *CIRCLE-seq* (260/6903) contained sequences spanning 22 or 24 nt, which indicates that these three techniques can detect OTS with DNA/RNA bulges (22) at the RNA-DNA interface. In our study, DNA/RNA bulges were not considered, therefore OTS spanning less or more than 23 nt were all excluded. In addition, OTS only with the canonical-PAM NGG was considered in our study for the spCas9 system. Then, we noticed that *CIRCLE-seq* (11/3106), *SITE-Seq* (27/70) and *PEM-seq* (1/52) detected a small portion of OTS with mismatches >6 bases compared to the gRNA targets at the first 20 nt before PAM. In our study, the maximum mismatch tolerance of OTS was set to 6. Finally, OTS spanning 23 nt with NGG-PAM and mismatches up to 6 bases were curated in our analysis, resulting in the curated datasets with 3905 OTS of 16 gRNAs from *CIRCLE-seq*, 109 OTS of 11 gRNAs from *Digenome-seq*, 38 OTS of 8 gRNAs from *DIG-seq*, 43 OTS of 6 gRNAs from *SITE-Seq*, 14 OTS of 2 gRNAs from *BLESS*, 209 OTS of 8 gRNAs from *GUIDE-seq*, 72 OTS of 4 gRNAs from *HTGTS*, 19 OTS of 4 gRNAs from *IDLV*, 51 OTS of 2 gRNAs from *PEM-seq*, and 2 OTS of 1 gRNA from *DISCOVER-Seq*. The curated OTS datasets were used to benchmark these OTS detection techniques. The details of the experimental techniques and the curated datasets are listed in Supplementary Table S1.

### Benchmark *in silico* genome-wide CRISPR OTS prediction tools

#### Categories of *in silico* CRISPR OTS prediction tools

Table 2 presents 17 available *in silico* tools for genome-wide CRISPR OTS prediction. These tools are categorized into four types: (i) alignment-based (23–31), in which the potential OTS on a given genome are searched purely based on sequence alignment to the intended target sequence with certain constraints; (ii) hypothesis-driven (32–35), in which the candidate OTS are predicted and scored with the contribution of specific sequence factors on off-target cleavage activity; (iii) learning-based (3,36), in which candidate OTS are predicted and scored based on a training model with features affecting the off-target efficacy and (iv) energy-based (37,38), in which candidate OTS are predicted and scored based on a free-energy model for Cas9–gRNA–DNA binding. A previous study by Alkan *et al.* indicated that the learning-based *Elevation* was more like a transformation of *CFD* (37). Therefore, we excluded *Elevation* in our follow-up benchmarking of prediction tools.

**Table 2.** List of four categories of *in silico* CRISPR OTS prediction tools benchmarked in our study

Category	Tool	OTS prediction	Multiple organism accessible	Reference
Alignment-based	Cas-OFFinder	mismatch	Yes	(24)
	CRISPR Finder	mismatch	Human (GRCh38) + mouse (GRCm38)	(26)
	E-CRISP	mismatch	Yes (GRCh38)	(25)
	CHOPCHOP	mismatch	Yes	(27)
	CRISPRscan	mismatch	Yes (GRCh38)	(28)
	sgRNAcas9	mismatch	Yes	(31)
	GT-Scan	mismatch	Yes	(29)
	Off-Spotter	mismatch	Yes	(30)
	CasFinder	mismatch	Yes	(23)
Hypothesis-driven	CFD	mismatch + score	Yes	(32)
	MIT	mismatch + score	Yes	(33)
	CCTop	mismatch + score	Yes	(34)
	CROP-IT	mismatch + score	Human (hg19) + mouse (mm9)	(35)
Learning-based	DeepCRISPR	mismatch + score	Human (hg19; 13 cell-types)	(36)
	Elevation	mismatch + score	Human (GRCh38)	(3)
Energy-based	CRISPR-OFF	mismatch + score	Yes	(37)
	uCRISPR	mismatch + score	Yes	(38)

#### Generation of benchmark datasets for *in silico* OTS prediction tools assessment

After an assessment of alignment-based OTS prediction tools, *Cas-OFFinder* was considered as the best choice for genome-wide candidate OTS searching (see Results). We aimed at generating the benchmark datasets based on OTS detection techniques for the following assessment of other three types of *in silico* OTS prediction tools. So, we first applied *Cas-OFFinder* to generate the genome-wide candidate OTS of tested gRNAs from the curated datasets generated before, restricting the off-target with 23-nt long, containing NGG-PAM and mismatches up to 6 bases. Then, among the candidate OTS, the validated OTS were labeled ‘1’, otherwise labeled ‘0’. Considering that the learning-based tool *DeepCRISPR* covers some published OTS datasets to train its learning model and currently supports OTS prediction in only 13 mainstream cell types, datasets trained by *DeepCRISPR* and gRNAs not detected in these 13 cell types were removed from the benchmark datasets for *in silico* OTS prediction tools assessment. Since the benchmark of experimental OTS detection techniques showed that the CRISPR cleavage specificity is heterogeneous in different cell types (see Results), the gRNAs in datasets were classified into three groups by their detection cell types, and the OTS on given gRNAs in same cell types shared by different techniques were merged together. Finally, this resulted in the benchmark datasets containing 444 921 candidates OTS with 1850 positive labels in 3 cell types, which includes 180 671, 76 059 and 188 191 candidates with 968, 778 and 104 positive labels from 12, 7 and 11 gRNAs respectively in HEK293, K562 and HeLa. The details of the benchmark datasets are listed in Supplementary Table S4.

### Implementation of iGWOS platform

#### Generation of train and test datasets for ensemble model by integrating CRISPR OTS prediction tools

The prediction results obtained from seven *in silico* OTS prediction tools on benchmark datasets were added as the OTS features to the benchmark datasets generated above, containing 444 921 candidates OTS with 1850 positive labels in three cell types (Supplementary Table S4). We extracted 80% from each cell type of the benchmark datasets as the train dataset and tuned model parameters with cross-validation. Then the left 20% was taken as an independent test dataset, used to test the performance of our trained model. The details of the train and test datasets are listed respectively in Supplementary Table S5 and Supplementary Table S6.

#### Train the AdaBoost ensemble model based on the train dataset

AdaBoost is a successful boosting algorithm developed for binary classification, which is best used with weak learners, like decision stump (decision trees with one level). It adds a weak learner in each iteration to learn misclassified training instances until a pre-set number of weak learners is created or no more improvement can be made on the train dataset. In this study, the AdaBoost model parameters were tuned on the train dataset under 5-fold stratified cross-validation (i.e., keep the class distribution in each fold almost identical to that in the original data), and the best parameters were selected, where algorithm was set to ‘SAMME.R’, base_estimator set to decision stump, n_estimators set to 280 and learning_rate set to 0.1. The predicted class ‘1’ probability for a candidate OTS was taken as the integrative prediction score *iGWOS*, denoting the cleavage probability of a candidate OTS.

#### Development of iGWOS platform

The *iGWOS* (**i**ntegrated **G**enome-**W**ide **O**ff-target cleavage **S**earch) platform is designed specifically for the optimal OTS prediction by integrating the available *in silico* OTS prediction tools with an Adaboost framework. *iGWOS* currently supports precise genome-wide CRISPR OTS prediction with conventional NGG-PAM and mismatches up to 6 bases in human species. By inputting the gRNA(s) sequence file and related restrictions at the command line, *iGWOS* outputs integrated prediction of the genome-wide OTS profile of given gRNAs and visualizes the top 200 risky genome-wide off-target profile with a *Circos* (39) plot. Details regarding the usage and installation of our ensemble package *iGWOS* can be referred on GitHub at https://github.com/bm2-lab/iGWOS.

## RESULTS

### Benchmark genome-wide CRISPR OTS detection

#### Categorizing experimental CRISPR OTS detection techniques

In the last few years, a couple of studies evaluated genome-wide CRISPR off-target detection, aiming to quantitatively analyze the specificity of the CRISPR system. These techniques were categorized into three types according to the DSB detection conditions, i.e. *in vitro*, cell-based and *in vivo* (Table 1). Although recent studies (4–6) reviewed several of these techniques with respect to their operating principles and operational protocols, further comprehensive and quantitative comparisons are still needed. Here we benchmarked these techniques on a genome-wide profile based on the publicly available OTS detection datasets generated from the corresponding techniques, aiming to present the first objective guidance for selection of genome-wide OTS detection techniques and the following benchmark of *in silico* OTS prediction tools. After screening, we obtained the curated datasets from 10 OTS detection techniques. Detailed information regarding the dataset curation is provided in the Materials and Methods section and Supplementary Table S1.

#### Benchmark result of experimental CRISPR OTS detection techniques

**Figure 2.**
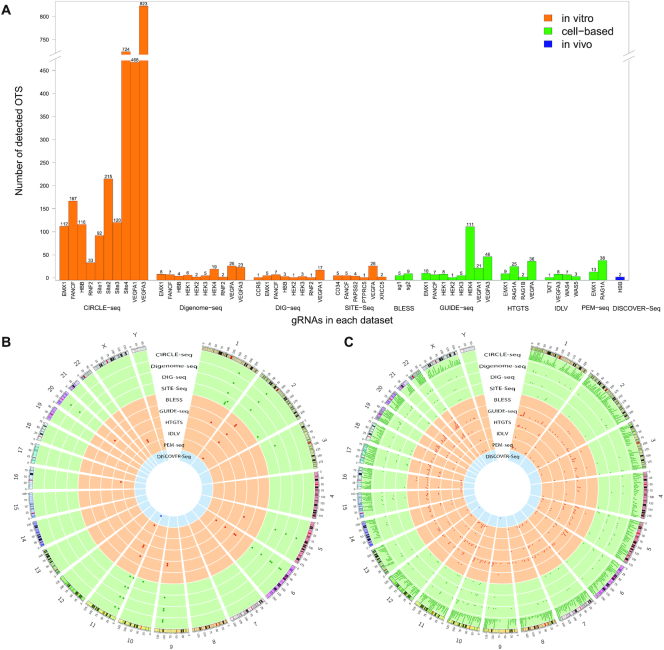
gRNA sequences and OTS distribution among the curated datasets corresponding to the 10 OTS detection techniques. (**A**) The number of detected genome-wide OTS in tested gRNA sequences. (**B**) The *Circos* plot shows the gRNA sequences on the reference genome (hg19). Ten tracks from the outer to inner parts of the plot represents the 10 curated datasets, in which each point represents an gRNA sequence. (**C**) The *Circos* plot shows the OTS distribution among the 10 curated datasets.

The overall comparison on the number of detected OTS in the publicly available tested gRNA sequences among the 10 curated datasets clearly indicated that *CIRCLE-seq* was the most sensitive technique among all three categories because it detected many more validated genome-wide OTS compared with the other techniques (Figure 2A). The *Circos* plot displayed gRNA sequences and the corresponding off-target sites of the 10 curated datasets on a genome-wide scale (Figure 2B and C). The gRNA sequences distribution indicated the overlaps of gRNA sequences shared among multiple datasets. So, the gRNA sequences that were commonly tested by several datasets were selected for further comparison (Supplementary Table S2).

**Figure 3.**
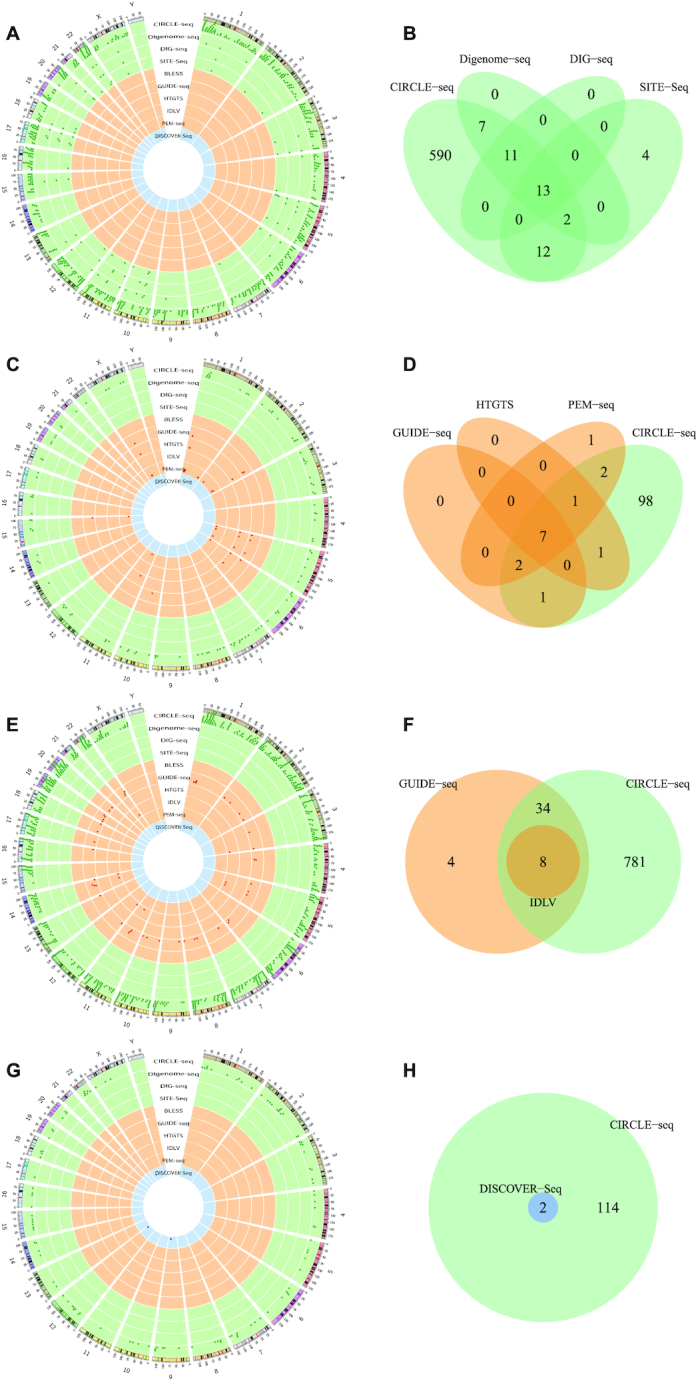
Genome-wide OTS distribution and intersections of tested gRNA sequences shared by multiple techniques. (**A** and **B**) OTS distribution and intersections of two tested gRNAs (‘VEGFA1’ and ‘FANCF’) shared by four *in vitro* techniques. (**C** and **D**) OTS distribution and intersections of tested gRNA ‘EMX1’ shared by three cell-based techniques and *in vitro CIRCLE-seq*. (**E** and **F**) OTS distribution and intersections of tested gRNA ‘VEGFA3’ shared by two cell-based techniques and *in vitro CIRCLE-seq*. (**G** and **H**) OTS distribution and intersections of tested gRNA ‘HBB’ shared by *in vivo DISCOVER-Seq* and *in vitro CIRCLE-seq*.

By comparing the genome-wide OTS distribution and the overlapping OTS of the gRNA sequences shared by four *in vitro* techniques, *CIRCLE-seq* was confirmed to be the most sensitive *in vitro* genome-wide OTS detection technique compared with the other *in vitro* techniques (Figure 3A and B). Similarly, the comparison of cell-based techniques indicated that all of these techniques detected a small portion of specific OTS not shared by the other cell-based techniques, and they were almost covered by the *in vitro CIRCLE-seq* (Figure 3C–F). In addition, the *in vivo* technique *DISCOVER-Seq* detected only a few OTS, and they were all covered by the *in vitro CIRCLE-seq* (Figure 3G and H). Taken together, we concluded that (i) *CIRCLE-seq* was the most sensitive OTS detection technique among the three OTS detection categories and (ii) OTS detection techniques have their unique characteristics, resulting from their different experimental categories, DSBs detecting sensitivities, and even the developing times.

**Figure 4.**
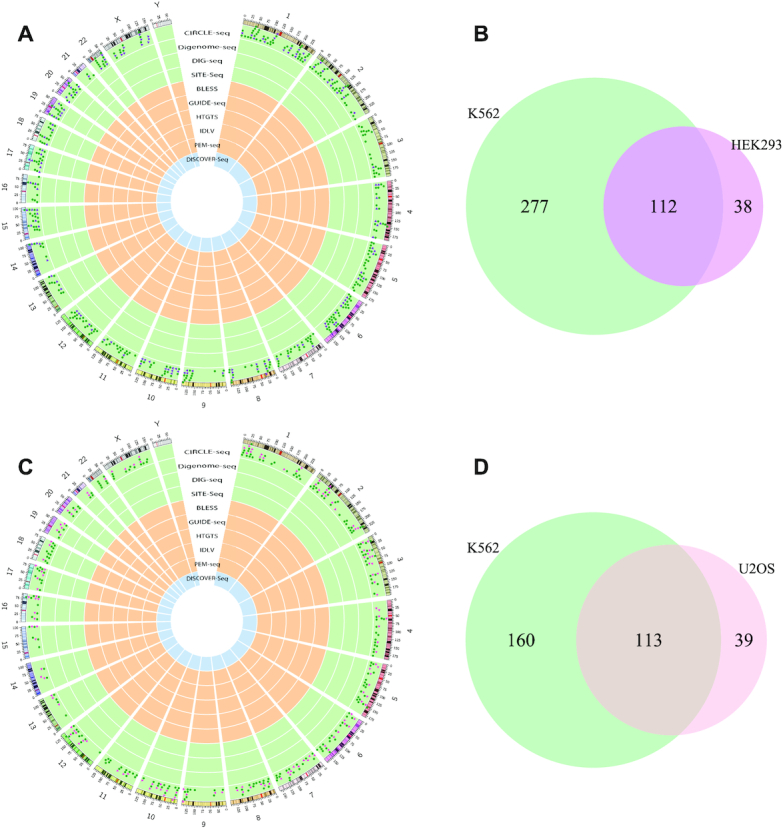
Genome-wide OTS distribution and intersections of the same gRNA sequences shared in different cell types detected by *CIRCLE-seq*. (**A** and **B**) OTS distribution and intersections of three tested gRNA sequences shared by K562 and HEK293. (**C** and **D**) OTS distribution and intersections of three tested gRNA sequences shared by K562 and U2OS.

After the comparison of gRNA sequences overlapped among multiple datasets, we also noticed some gRNA sequences were tested in different cell types in the curated dataset of *CIRCLE-seq* (Supplementary Table S2). By comparing the OTS distribution and intersections of same gRNA sequences shared in different cell types, we found that both K562 and HEK293 could a portion of specific OTS not shared by the other, and K562 detected much more OTS than those in HEK293 (Figure 4A and B). The similar result was obtained when comparing the OTS intersections of same gRNA sequences detected in K562 and U2OS (Figure 4C and D). Taken together, we concluded that (i) the CRISPR cleavage specificity is heterogeneous in different cell types, likely resulting from their different genetic and epigenetic information and (ii) some cell types such as K562 tend to generate much more off-target cleavages in CRISPR knock-out experiments.

In summary, the benchmark result of experimental CRISPR OTS detection techniques shows that three categories of experimental OTS detection techniques showed their own characteristics in OTS detection and the specificities of given gRNAs verifies in different cell types.

In the following benchmark of *in silico* CRISPR OTS prediction tools, the curated dataset generated from OTS detection techniques were used to generate the benchmark datasets to assess the performances of the available *in silico* genome-wide OTS prediction tools.

### Benchmark genome-wide CRISPR OTS prediction

#### Categorizing *in silico* CRISPR OTS prediction tools

Numerous *in silico* tools have been presented for predicting CRISPR-Cas9 OTS in human species. These tools are categorized into four types according to the OTS prediction mechanism, i.e. alignment-based, hypothesis-driven, learning-based and energy-based (Table 2). A previous study by Haeussler *et al.* evaluated four hypothesis-driven off-target prediction algorithms (2), but comprehensive comparison and assessment of all categories of OTS prediction algorithms have not yet been performed. Here, we present an objective and comprehensive comparison of all these algorithms based on the benchmark datasets generated from the curated datasets, offering a reliable recommendation and selection for *in silico* OTS prediction in various scenarios. Detailed characteristics of these tools are presented in Supplementary Table S3.

#### Benchmark results of *in silico* CRISPR OTS prediction tools

##### Benchmark alignment-based tools for candidate OTS searching

As alignment-based tools predict candidate OTS purely by sequence alignment without ranking their potential knockout ability, we benchmarked this type of tool based on their options for maximum candidate off-target searching (Table 3). The table illustrated that *Cas-OFFinder* shows its advantage in genome-wide candidate OTS searching compared with other alignment-based tools, with unlimited mismatch tolerance and supporting for batch searching offline. Therefore, in the following benchmark of the other three categories of *in silico* OTS prediction tools, *Cas-OFFinder* was applied to obtain the genome-wide candidate OTS with NGG-PAM and mismatches up to six for tested gRNAs in the curated datasets to generate the benchmark datasets (see Materials and Methods).

**Table 3.** Benchmark alignment-based *in silico* CRISPR OTS prediction tools by their options for candidate off-target searching

Tool	Batch search	Running time	Mismatch tolerance	PAM pattern	Platform
Cas-OFFinder	Yes	Medium	Not limited	NRG (R = A or G)	Web/Online/Graphic interface + C++/Offline/Command-line
CRISPR Finder	No (Web Crawler)	Medium	4	NGG	Web/Online/Graphic interface
E-CRISP	Yes	Medium	3	NRG	Web/Online/Graphic interface
CHOPCHOP	No (Web Crawler)	Fast	3	NRG; NGA	Web/Online/Graphic interface
CRISPRscan	Yes	Fast	4	NGG	Web/Online/Graphic interface
sgRNAcas9	Yes	Slow	5	NRG	Perl/Offline/Command-line + Java/GUI
GT-Scan	Yes	Fast	3	NRG	Python/Offline/Command-line
Off-Spotter	No (Web Crawler)	Fast	5	NGG	Web/Online/Graphic interface
CasFinder	Yes	Fast	3	NRG	Python/Offline/Command-line

##### Benchmark *in silico* OTS prediction tools with the benchmark datasets

**Figure 5.**
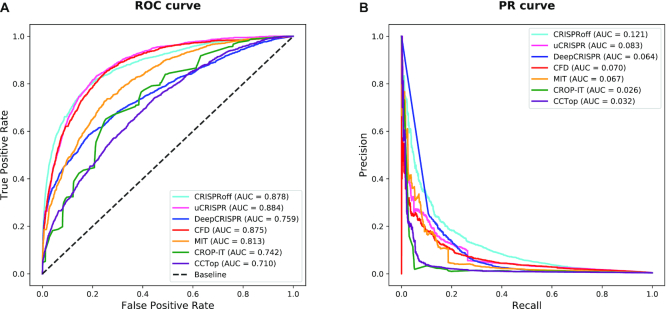
Benchmark of OTS prediction performances of hypothesis-driven, learning-based, and energy-based tools with the benchmark datasets.

To assess the hypothesis-driven, learning-based, and energy-based OTS prediction tools based on the benchmark datasets (see Materials and Methods and Supplementary Table S4), the prediction scores of candidate OTS in benchmark datasets were calculated from individual tools. Considering that a majority of the benchmark datasets were from the negative class and precision-recall (PR) curve is more sensitive to class imbalance than the receiver operating characteristic (ROC) curve, both the ROC curve and PR curve were used to evaluate the prediction performances of these tools (Figure 5). The assessment showed that (1) the energy-based tools performed higher ROC-area under the curve (AUC) (0.878 for *CRISPRoff*, and 0.884 for *uCRISPR*) and higher PR-AUC (0.121 for *CRISPRoff*, and 0.083 for *uCRISPR*) than the hypothesis-driven tools, showing their better ability to predict OTS, (2) *DeepCRISPR* did not show excellent performance, likely resulting from the data-driving limitation for learning-based tools and the testing data in this study is different from previous study (38), and (3) *CFD* performed the best among the hypothesis-driven tools. In summary, both the ROC–AUC and PR-AUC values indicated that the structural and energy-based mechanism of CRISPR binding helps to improve OTS prediction compared with the hypothesis-driven and learning-based ones.

### iGWOS: integrated Genome-Wide Off-target cleavage Search

Our benchmark above indicated that each categories of prediction tools has its own characteristic in OTS perdition, and an effective integration of those OTS prediction tools may contribute to a better performance in genome-wide OTS prediction. A recent study also showed that synergizing multiple hypothesis-driven tools with an ensemble learning method enhanced OTS prediction (40). Therefore, we attempted to combine the OTS prediction results obtained from individual benchmarked tools using the ensemble framework AdaBoost (41) to improve the performance of OTS prediction.

**Figure 6.**
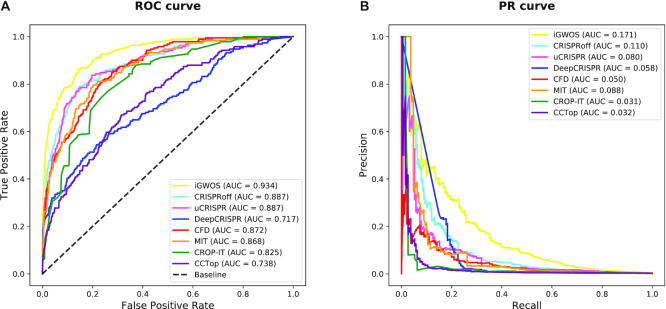
ROC and PR curves of *iGWOS* compared to individual *in silico* tools in genome-wide OTS prediction with the test dataset.

The benchmark datasets for ensemble model took the prediction scores from 7 prediction algorithms as the OTS features, and then were split into train dataset and test dataset (see Materials and Methods). The AdaBoost ensemble model was trained on the train dataset under a 5-fold stratified cross-validation to tune the model parameters. Finally, the prediction performances on the test dataset showed that our trained model *iGWOS* outperformed the existing individual tools, providing a substantial improvement in genome-wide OTS prediction (Figure 6).

Finally, a one-stop **i**ntegrated **G**enome-**W**ide **O**ff-target cleavage **S**earch platform, i.e., *iGWOS* was developed that is available on GitHub at https://github.com/bm2-lab/iGWOS, which was designed to precisely predict genome-wide CRISPR OTS profiles by integrating three categories of OTS prediction algorithms using an AdaBoost framework.

## DISCUSSION

The off-target effect of the CRISPR/Cas9 system remains to be an obstacle for successful therapeutic application of genome editing. Therefore, many techniques and tools have been proposed or developed to better detect and predict genome-wide OTS in different environments. Our comprehensive benchmark study of these existing resources provides insightful guidance for off-target effect research in four aspects: (i) The benchmarking of experimental CRISPR off-target detection techniques indicated that the gRNA specificity verifies in different experimental OTS detection techniques, resulting from their different experiment categories, DSBs detecting sensitivities, and even the developing times. A recent study provided a new *in vitro* genome-wide OTS technique called CHANGE-seq (42), which was reported to perform better than *CIRCLE-seq* in sequencing efficacy and parallel experiments. (ii) CRISPR cleavage specificity is heterogeneous in different cell types, resulting from their different genetic and epigenetic information. (iii) The structural and energy-based mechanisms of CRISPR binding, taking the characteristics of DNA-RNA binding into account and without requiring a large amount of training data, generally contribute to a better performance in genome-wide OTS prediction, which will promote further researches of CRISPR off-target effect based on the structural mechanisms and molecular modeling. (iv) The development of our *iGWOS* platform confirmed that the integration of different categories of prediction algorithms is an efficient strategy for achieving better off-target prediction.

## DATA AVAILABILITY

The authors declare that the datasets and results discussed in this study are available within the article and its supplementary information files. Besides, the source code of *iGWOS* platform is also available under an open source license (GNU General Public License v3.0) at GitHub.

## Supplementary Material

gkaa930_Supplemental_Files
