# Supplementary material for: Benchmarking and integrating genome-wide CRISPR off-target detection and prediction
Source: Nucleic Acids Res. 2020 Nov 2;48(20):11370–9. doi: 10.1093/nar/gkaa930 (PMC7672467; doi:10.1093/nar/gkaa930)
Supplement: gkaa930_Supplemental_Files [file gkaa930_supplemental_files.zip › Supplementary Materials Legends.docx]

**Supplementary information**

Supplementary Table 1-5

Supplementary Table 1: Summary of genome-wide off-target detection techniques benchmarked in our study.

Supplementary Table 2: gRNAs shared by several OTS detection datasets and gRNAs shared in different cell types detected by CIRCLE-seq.

Supplementary Table 3: Summary of OTS prediction tools benchmarked in our study.

Supplementary Table 4: The benchmark datasets for prediction tools assessment.

Supplementary Table 5: The train dataset for ensemble model training.

Supplementary Table 6: The test dataset for ensemble model assessment.
